# SGLT2 inhibition, high-density lipoprotein, and kidney function: a mendelian randomization study

**DOI:** 10.1186/s12944-024-02072-6

**Published:** 2024-03-20

**Authors:** Zhijuan Wang, Jie Wei, Wenman Zhao, Rui Shi, Yuyu Zhu, Xunliang Li, Deguang Wang

**Affiliations:** 1 Department of Nephrology, the Second Affiliated Hospital of Anhui Medical University, 678 Furong Road, Hefei, 230601 Anhui China; 2 Institute of Kidney Disease, Inflammation & Immunity Mediated Diseases, the Second Affiliated Hospital of Anhui Medical University, 678 Furong Road, Hefei, 230601 Anhui China

**Keywords:** Sodium-glucose cotransporter 2 inhibition, High-density lipoprotein, Kidney function, Mendelian randomization

## Abstract

**Background:**

Sodium-glucose cotransporter 2 (SGLT2) inhibition is recognized for its evident renoprotective benefits in diabetic renal disease. Recent data suggest that SGLT2 inhibition also slows down kidney disease progression and reduces the risk of acute kidney injury, regardless of whether the patient has diabetes or not, but the mechanism behind these observed effects remains elusive. The objective of this study is to utilize a mendelian randomization (MR) methodology to comprehensively examine the influence of metabolites in circulation regarding the impact of SGLT2 inhibition on kidney function.

**Methods:**

We used a MR study to obtain associations between genetic proxies for SGLT2 inhibition and kidney function. We retrieved the most recent and comprehensive summary statistics from genome-wide association studies (GWAS) that have been previously published and involved kidney function parameters such as estimated glomerular filtration rate (eGFR), urine albumin-to-creatinine ratio (UACR), and albuminuria. Additionally, we included blood metabolite data from 249 biomarkers in the UK Biobank for a more comprehensive analysis. We performed MR analyses to explore the causal relationships between SGLT2 inhibition and kidney function and two-step MR to discover potential mediating metabolites.

**Results:**

The study found that a decrease in HbA1c levels by one standard deviation, which is genetically expected to result in SGLT2 inhibition, was linked to a decreased likelihood of developing type 2 diabetes mellitus (T2DM) (odds ratio [OR] = 0.55 [95% CI 0.35, 0.85], *P* = 0.007). Meanwhile, SGLT2 inhibition also protects eGFR (β = 0.05 [95% CI 0.03, 0.08], *P* = 2.45 × 10^− 5^) and decreased UACR (-0.18 [95% CI -0.33, -0.02], *P* = 0.025) and albuminuria (-1.07 [95% CI -1.58, -0.57], *P* = 3.60 × 10^− 5^). Furthermore, the study found that of the 249 metabolites present in the blood, only one metabolite, specifically the concentration of small high-density lipoprotein (HDL) particles, was significantly correlated with both SGLT2 inhibition and kidney function. This metabolite was found to play a crucial role in mediating the improvement of renal function through the use of SGLT2 inhibition (β = 0.01 [95% CI 0.005, 0.018], *P* = 0.001), with a mediated proportion of 13.33% (95% CI [5.71%, 26.67%], *P* = 0.020).

**Conclusions:**

The findings of this investigation provide evidence in favor of a genetically anticipated biological linkage between the inhibition of SGLT2, the presence of circulating metabolites, and renal function. The findings demonstrate that the protective effect of SGLT2 inhibition on renal function is mostly mediated by HDL particle concentrations in circulating metabolites. These results offer significant theoretical support for both the preservation of renal function and a better comprehension of the mechanisms underlying SGLT2 inhibition.

**Supplementary Information:**

The online version contains supplementary material available at 10.1186/s12944-024-02072-6.

## Introduction

Sodium-glucose cotransporter 2 (SGLT2) inhibition is a new type of antihyperglycemic medication that regulates glucose homeostasis by blocking the reabsorption of glucose in the proximal renal tubules of the kidney [1]. Based on nearly a decade of preclinical studies, SGLT2 inhibition has demonstrated a number of compelling benefits. In the kidney, this drug acts primarily by restoring abnormal tubulo-glomerular feedback and lowering intraglomerular pressure. In addition, SGLT2 inhibition has been shown to possess systemic metabolic and hemodynamic modulation, mitigation of mitochondrial dysfunction, reduction of oxidative stress and inflammatory responses, and promotion of autophagy [2]. Although several clinical studies have demonstrated the importance of SGLT2 inhibition in the protection of renal function, studies on their specific molecular mechanisms remain scarce, especially in non-diabetic patient populations.

According to recent research evidence, the link between metabolic reprogramming and kidney disease may have a profound impact on disease progression and patient prognosis [3, 4]. The metabolic-regulating effects of SGLT2 inhibition have been demonstrated; however, further investigation is required to elucidate the precise underlying mechanism [5]. Studies have shown that SGLT2 inhibition is able to directly regulate metabolic processes, thereby lowering blood glucose levels and affecting insulin and glucagon secretion as well as endogenous glucose production [6]. In experiments with diabetic mice, iglitazone treatment significantly reversed the accumulation of tricarboxylic acid cycle intermediates and increased oxidative stress in the renal cortex, which was consistent with an improvement in glomerular injury [7]. In addition, lipid responses, including lipolysis, oxidation, and ketogenic processes, were enhanced after long-term use of SGLT2 inhibition. This phenomenon was seen in both diabetic and nondiabetic patients, suggesting a shift in fuel utilization from carbohydrates to fatty acids and ketone bodies [8]. These findings reveal the potential role of metabolic disorders in kidney disease and suggest that SGLT2 inhibition may protect kidney function by ameliorating metabolic disorders.

The mendelian randomization (MR) study, a powerful tool in epidemiological research, utilizes genetic variation as an effective means of assessing causal relationships between risk factors and specific diseases [9]. In this study, genetic variation follows the principle of assigning alleles to offspring, similar to randomized controlled experiments. This approach significantly reduces confounding by confounding factors and reverse causation, which are common in observational studies [10]. Given the fact that the metabolic mechanisms of SGLT2 inhibition in preventing renal function have not been fully explored and the important role of metabolic pathways in the pathogenesis of renal disease, it is necessary to delve deeper into the mechanisms of action. In light of the above information, we have conducted a two-step MR study to investigate the causal relationship between circulating metabolites and the influence of SGLT2 inhibition on renal function. The present investigation is anticipated to yield significant insights regarding the underlying mechanism by which SGLT2 inhibition effectively mitigates the risk of kidney disease.

## Research Design and methods

### Study Design

**Fig. 1:**
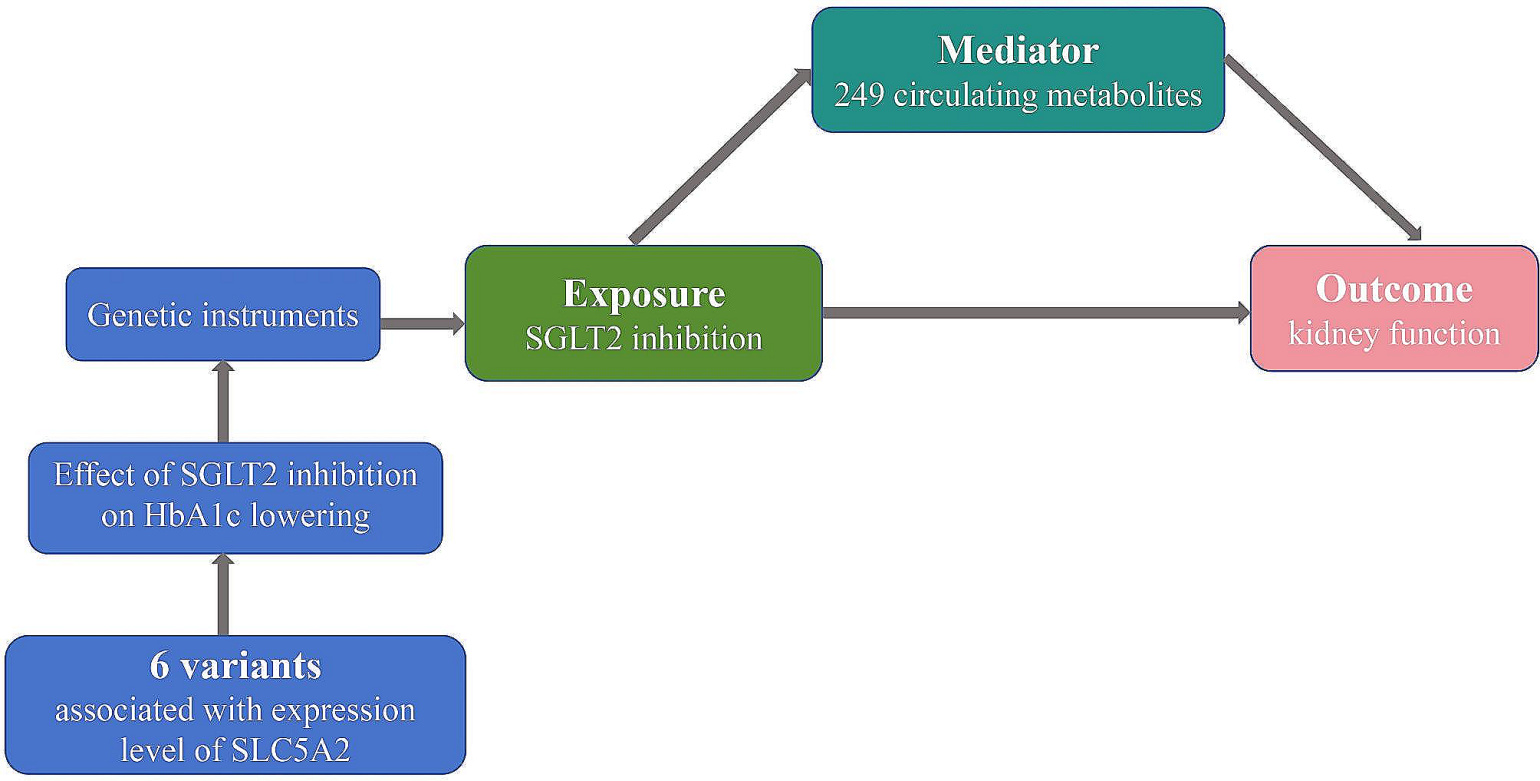
Flow Diagram of this study SGLT2: sodium-glucose cotransporter 2; HbA1c: glycated hemoglobin

Figure 1 depicts the research design schematic. The study encompasses four key stages:1) The identification of genetic variations that can act as indicators for the effects of SGLT2 inhibition. 2) The selection of 249 metabolites in circulation as potential intermediaries. 3) The selection of three kidney function outcomes: estimated glomerular filtration rate (eGFR), urinary albumin-to-creatinine ratio (UACR), and albuminuria. 4) This study employs a two-step MR technique to estimate the causal effects of SGLT2 inhibition and circulating metabolites on kidney function. Additionally, it aims to evaluate the mediating role of circulating metabolites in the association between SGLT2 inhibition and renal function outcomes.

### The process of selecting and validating genetic predictors of SGLT2 inhibition

The approach to selecting genetic variations as a proxy for SGLT2 inhibition consisted of four distinct steps. Initially, genetic variants associated with the mRNA expression level of the SLC5A2 gene were identified using data from the Genotype-Tissue Expression (GTEx) project [11] and the eQTLGen Consortium [12], along with information on the potential functional genes of SGLT2 inhibition. Subsequently, the association of glycated hemoglobin level (HbA1c) with each SLC5A2 variant was estimated. HbA1c is a measure of glucose-lowering effect via SGLT2 inhibition. The present research was performed utilizing data obtained from a subset of unrelated individuals of European descent who do not have diabetes, sourced from the UK Biobank (*n* = 344,182), with an association *P* value set at 1 × 10^− 4^ [13, 14]. The genetic colocalization technique was utilized to ascertain whether there is a shared causal variation between SLC5A2 and HbA1c. The present study utilizes a bivariable genetic strategy that employs a Bayesian model for the purpose of estimating the posterior probability that the two features, namely the expression of SLC5A2 and the circulating HbA1c level, are influenced by the same causal variant located within the SLC5A2 region [15]. Colocalization between the expressions of SLC5A2 and HbA1c was deemed to be present when the colocalization probability exceeded 70%. Finally, after completing the selection and validation procedures, a standard clumping technique was employed to exclude variations exhibiting an exceedingly high correlation (using a correlation threshold of < 0.8).

### Genetic instruments for circulating metabolites

A thorough investigation was carried out on a total of 249 circulating metabolites obtained using nuclear magnetic resonance analysis. These metabolites were derived from a sample size of 121,000 individuals of European ancestry. The data was collected and provided by Nightingale Health [16]. The dataset of metabolic biomarkers consisted of 168 absolute concentrations and 81 ratios. In the MR analysis, absolute concentrations of 249 biomarkers were utilized. The genome-wide association studies (GWAS) summary statistics for these biomarkers were publicly released through the IEU Open-GWAS Project database, identified by the GWAS identifier ‘met-d’. We limited our analysis to genetic variants that were both genome-wide significant (with a *P*-value less than 5 × 10^− 8^) and independent of each other (with a linkage disequilibrium r^2^ value less than 0.01 within a 10,000 kb region).

### Study outcomes

The meta-analysis of the GWAS of eGFR encompassed 54 cohorts of individuals of European ancestry, totaling 567,460 participants. eGFR was calculated using the Chronic Kidney Disease Epidemiology Collaboration equation for adults over the age of 18 [17], and the Schwartz formula for individuals under the age of 18 [18]. The UACR data were derived from a meta-analysis that compiled summary data across multiple ethnic groups (*n* = 564,257) and individuals of European ancestry [19]. The GWAS summary statistics for albuminuria were obtained from a meta-analysis of GWAS (*n* = 348,954), which adjusted for factors such as age, sex, study-specific covariates, and genetic principal components [19]. The summary statistics pertaining to type 2 diabetes mellitus (T2DM) were obtained from the Diabetes Genetics Replication and Meta-analysis (DIAGRAM) consortium. For this work, we obtained genome-wide association study (GWAS) data specific to individuals of European ancestry, with a sample size of 933,972 [20].

### Statistical analysis

#### Genetic analyses to elucidate causality

We conducted MR analysis to explore the relationship between SGLT2 inhibition and kidney function and T2DM. To establish a statistically significant causal link, we employed five distinct MR methods: random-effects inverse-variance weighted (IVW), MR Egger, weighted median, simple mode, and weighted mode. The IVW method was employed as the principal analytical approach, yielding the most accurate and reliable estimations when all genetic variants are considered legitimate instruments [21].

#### Mediation analyses link “SGLT2 inhibition–blood metabolites–eGFR”

We adopted two-step MR (TSMR) to decompose the direct and indirect effects of SGLT2 inhibition and blood metabolites on kidney function. In addition to the basic effect estimates of SGLT2 inhibition on blood metabolites (β1) obtained from the univariate MR analyses, two more estimates were calculated: the causal effect of the mediator (blood metabolites) on eGFR (β2) and the total effect of SGLT2 inhibition on eGFR (α). The indirect effect, which refers to the causal effect of SGLT2 inhibition on eGFR via mediators, can then be estimated using the product of coefficients method (β1 × β2). Thus, the proportion mediated could be calculated as “indirect effect/total effect” ([β1 × β2]/α). The confidence intervals (CIs) for the mediation proportions were computed using the delta approach with a 95% confidence level [22].

#### Validation of MR assumptions and sensitivity analyse

The study findings have been reported in accordance with the STROBE-MR guidelines, which aim to strengthen the reporting of MR studies. To test the three critical assumptions of MR, a set of sensitivity analyses was conducted. The relevance assumption was validated by estimating the strength of the genetic predictors using R2 and F-statistics. An F-statistic above 10 was considered evidence against weak instrument bias. The exclusion restriction assumption was tested using several sensitivity analyses, including MR-Egger regression, weighted median analysis, and simple and weighted mode analyses. Cochran’s Q test was utilized to assess heterogeneity between instruments [23].

All MR analyses were conducted in R (version 4.2.3) using “data.table,” “TwoSampleMR,” “ggplot2,” and “tidyverse.” A two-sided *P*-value that passed the Bonferroni-corrected *P*-value was considered statistically significant.

## Results

### Effect of SGLT2 inhibition on kidney function and T2DM

**Fig. 2:**
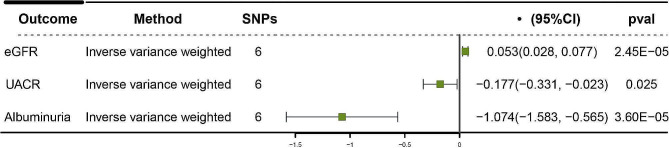
Causal effect of SGLT2 inhibition on kidney function SGLT2: sodium-glucose cotransporter 2; eGFR: estimated glomerular filtration rate; UACR: urinary albumin-to-creatinine ratio; SNP: single-nucleotide polymorphism; CI: confidence interval

In total, a set of six distinct single nucleotide polymorphisms (SNPs) were chosen as genetic instruments for the purpose of inhibiting SGLT2. It is noteworthy that all of these SNPs exhibited F-statistics exceeding a value of 10. In the analysis conducted, it was observed that the inhibition of SGLT2 was linked to a decreased risk of T2DM (OR = 0.55 [95% CI 0.35, 0.85], *P* = 0.007). In addition, findings on renal function showed that SGLT2 inhibition protected eGFR (β = 0.05 [95% CI 0.03, 0.08], *P* = 2.45 × 10^− 5^). Similarly, SGLT2 inhibition reduced UACR (-0.18 [95% CI -0.33,-0.02], *P* = 0.025) and albuminuria (-1.07 [95% CI -1.58,-0.57], *P* = 3.60 × 10^− 5^) levels (Fig. 2). The Cochran Q test for IVW and the MR-Egger intercept term both indicated that there was little evidence of heterogeneity or directional pleiotropy.

### Mediation analyses of potential circulating metabolites

**Fig. 3:**
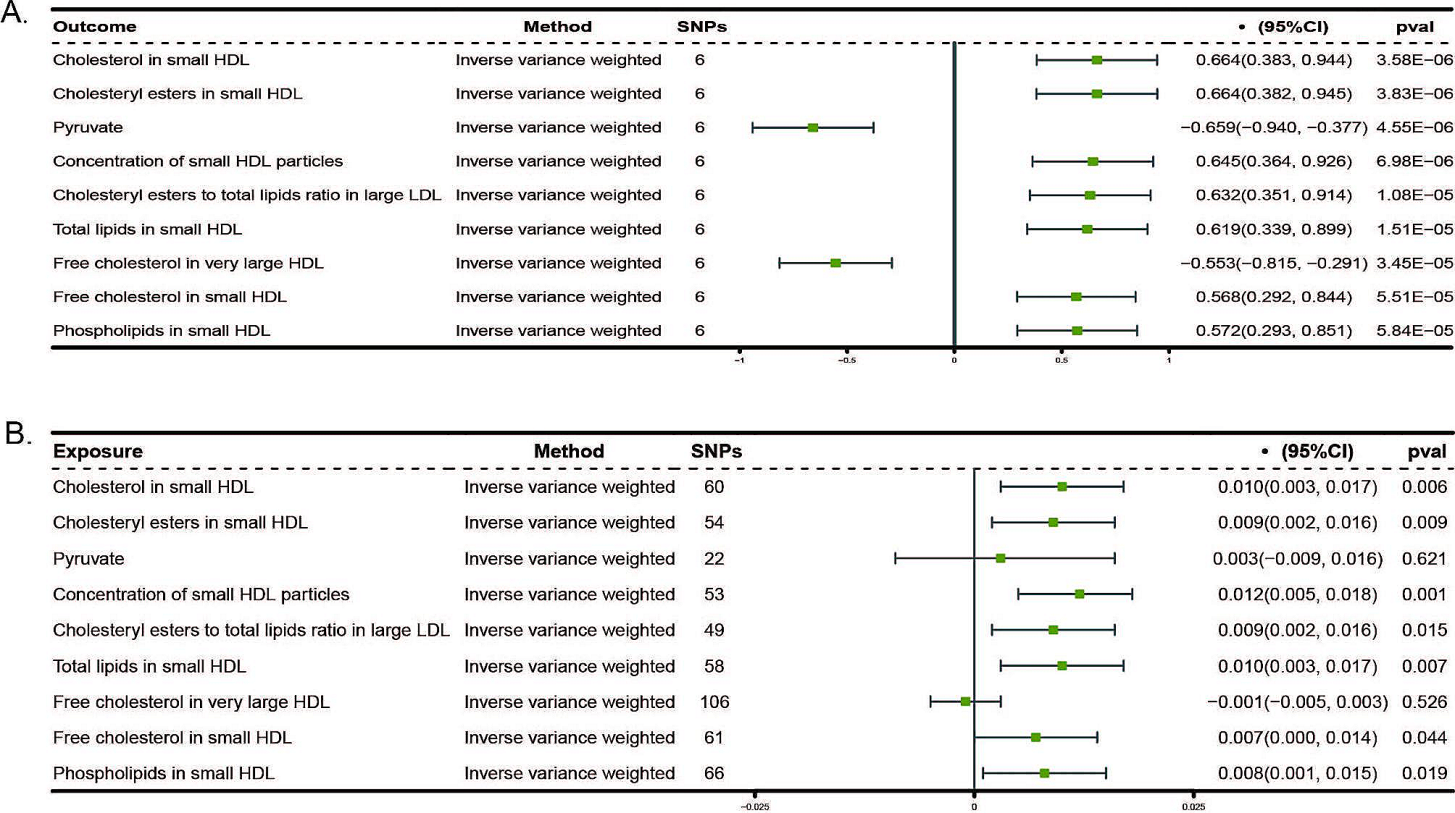
The causal effect of SGLT2 inhibition on circulating metabolites and the effect of metabolites on eGFR (**A**) The effect of SGLT2 inhibition on the remaining 9 circulating metabolites selected from 249 metabolites, which were significantly associated with SGLT2 inhibition (Bonferroni-corrected *P* value threshold = 2.00 × 10^− 4^ [0.05/249]) (**B**) The effect of the above 9 metabolites on eGFR (Bonferroni-corrected *P* value threshold = 0.0055 [0.05/9]) SGLT2: sodium-glucose cotransporter 2; eGFR: estimated glomerular filtration rate; HDL: high-density lipoprotein; LDL: low-density lipoprotein; SNP: single-nucleotide polymorphism; CI: confidence interval

Our analysis revealed that there were nine metabolites that exhibited a significant correlation with SGLT2 inhibition (Fig. 3A). To account for multiple comparisons, we applied a Bonferroni correction with a *p*-value threshold of 2.00 × 10^− 4^ [0.05/249]. These findings were based on our estimation of the impact of SGLT2 inhibition on 249 circulating metabolites. We observed that SGLT2 inhibition increased the total lipids in small high-density lipoprotein (HDL) (0.62 [95% CI 0.34, 0.90], *P* = 1.51 × 10^− 5^), cholesterol in small HDL (0.66 [95% CI 0.38, 0.94], *P* = 3.58 × 10^− 6^), cholesteryl esters in small HDL (0.66 [95% CI 0.38, 0.95], *P* = 3.83 × 10^− 6^), concentration of small HDL particles (0.65 [95% CI 0.36, 0.93], *P* = 6.98 × 10^− 6^), cholesteryl esters to total lipids ratio in large Low-density lipoprotein (LDL) (0.63 [95% CI 0.35, 0.91], *P* = 1.08 × 10^− 5^), free cholesterol in small HDL(0.57 [95% CI 0.29, 0.84], *P* = 5.51 × 10^− 5^),and phospholipids in small HDL(0.57[95% CI 0.29, 0.85], *P* = 5.84 × 10^− 5^). But decreased the pyruvate (-0.66 [95% CI -0.94, -0.38], *P* = 4.55 × 10^− 6^), and free cholesterol in very large HDL (-0.55 [95% CI -0.82, -0.29], *P* = 3.45 × 10^− 5^).

**Fig. 4:**
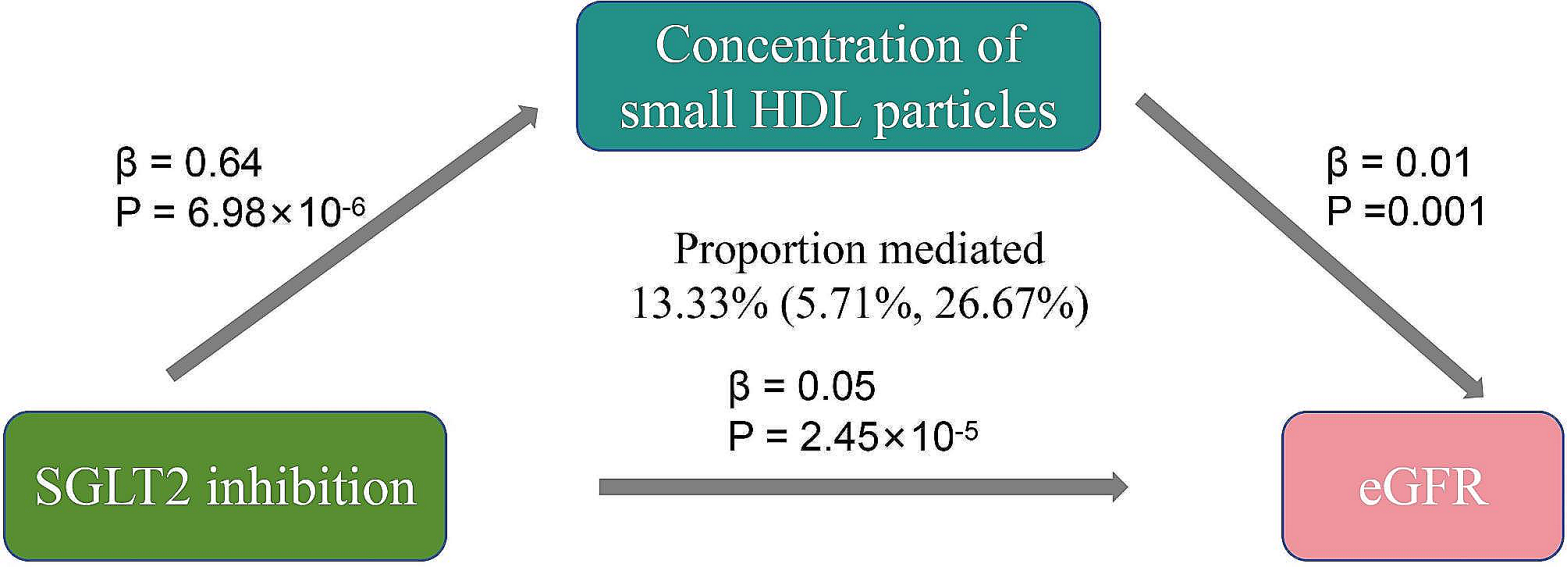
The potential causal evidence summarized from the MR analysis SGLT2: sodium-glucose cotransporter 2; eGFR: estimated glomerular filtration rate; HDL: high-density lipoprotein; MR: Mendelian Randomization

We conducted a further analysis to estimate the impact of the nine circulating metabolites that demonstrated a significant correlation with SGLT2 inhibition on the eGFR. Our findings indicate that one of these metabolites is significantly associated with eGFR, with a Bonferroni-corrected *P* value threshold of 0.0056 (accounting for the total number of metabolites examined, *n* = 9) (Fig. 3B). For the concentration of small HDL particles, we observed a positive association with eGFR (β = 0.01 [95% CI 0.005, 0.018], *P* = 0.001). The analysis revealed heterogeneity (Q = 486.596, *P* = 7.03 × 10^− 72^); however, there was no evidence of horizontal pleiotropy, as indicated by the Egger intercept (intercept = -0.0004, *P* = 0.208). The genetic variations associated with the nine metabolites exhibited significant strength, as shown by F statistics exceeding 10. An indirect effect of SGLT2 inhibition on eGFR was seen in our study. This effect was mediated through the concentration of small HDL particles, with a coefficient of 0.01 (95% CI 0.005, 0.018) and a significance level of *P* = 0.001 (Fig. 4). The mediated proportion of this indirect effect was estimated to be 13.33% (95% CI [5.71%, 26.67%]), with a significance level of *P* = 0.020, indicating a statistically significant contribution of small HDL particles to the overall effect.

## Discussion

The findings of our study suggest that there is a correlation between genetic diversity in SGLT2 inhibition targets and a reduced susceptibility to T2DM. Meanwhile, SGLT2 inhibition also protects eGFR and decreases UACR and albuminuria. Actually, the concentration of small HDL particles might mediate 13.33% of the effect of SGLT2 inhibition on eGFR.

In prior research on SGLT2 inhibition, investigators have primarily concentrated on its protective impact in specific areas, including diabetes, heart failure, and diabetic nephropathy. However, comprehensive investigations into the effects of SGLT2 inhibition on renal function in non-diabetic individuals are limited. If its renoprotective mechanism is clarified at the theoretical level, it will help to explore whether SGLT2 inhibition can be used as novel renoprotective drugs to stop or slow down the progression of chronic kidney disease and to promote clinical application in patients with non-diabetic nephropathy. Recently, a comprehensive meta-analysis of placebo-controlled SGLT2 inhibition demonstrated significant renal protective effects. Specifically, these drugs reduced the risk of kidney disease progression by 37% and the risk of acute kidney injury by 23%. This beneficial impact was observed in both non-diabetic and diabetic patients [24]. The study utilized MR research methods to minimize confounding factors. Furthermore, the study showed that the SGLT2 inhibitor gene surrogate predicted at the genetic level had a positive effect on renal function. This finding aligns with current guidelines for the use of antidiabetic agents in chronic kidney disease and evidence from randomized controlled trials [24, 25]. Therefore, this study provides robust evidence for the role of SGLT2 inhibition in preserving renal function in non-diabetic patients and enriches the scientific understanding in this field.

SGLT2 inhibition has multiple mechanisms of action in protecting renal function, including hemodynamic, metabolic, anti-inflammatory, and mitochondrial function regulation. These inhibitors increase urinary glucose excretion by acting on SGLT2 channels in the renal proximal tubular convoluted portion of the kidney, which may affect local or systemic metabolite changes [26]. Correlative studies have shown that SGLT2 inhibition is able to reduce renal injury and decrease the accumulation of toxic lipid metabolites (e.g., diacylglycerol, ceramides, and fatty acyl-coenzyme A) in podocytes, chloroplasts, and renal proximal tubules by inducing systemic glucose-to-fatty acid conversion. This process helps to reduce endoplasmic reticulum stress, oxidative stress, and inflammatory and fibrotic responses [1].

There are varying results from past studies on the lipid effects of SGLT2 inhibition. While a recent retrospective study found a decrease in LDL-C levels among diabetic patients newly treated with dagliflozin, most clinical studies with SGLT2 inhibition have demonstrated a slight but significant increase in LDL-C levels [27, 28]. Additionally, a small prospective multicenter study including 22 patients with T2DM showed an increase in mega HDL lipoproteins and large HDL lipoproteins of 10.9% and 11.5%, respectively, after 12 weeks of cagliflozin treatment [29]. Furthermore, both clinical and preclinical studies of SGLT2 inhibition have reported reductions in triglyceride levels [30]. In summary, these findings suggest that SGLT2 inhibition has the potential to correct abnormal lipid changes. However, these lipid changes may only be ancillary mechanisms by which SGLT2 inhibition exerts renal benefits. In this study, we utilized a MR approach to analyze how SGLT2 inhibition may affect renal function through modulation of circulating metabolites, particularly HDL. The findings of this study offer significant scientific insights into the possible impact of SGLT2 inhibition on the association between circulating metabolites and renal function.

The findings of our study indicate that SGLT2 inhibition significantly enhance HDL concentrations, particularly the smaller HDL subclasses, but also reduce the levels of large HDL and pyruvate. Furthermore, our genetic data provide evidence that HDL may mediate the protective effects of SGLT2 inhibition on renal function. Previous studies have found that an association between HDL cholesterol of different particle sizes and the risk of all-cause mortality in patients with end-stage diabetes. Specifically, while total HDL-C showed a U-shaped relationship with all-cause mortality in patients with T2D, small-particle HDL-C showed a U-shaped relationship with all-cause mortality, but cholesterol in small HDL showed an inverse linear relationship with all-cause mortality and cardiovascular disease mortality, whereas cholesterol in extra-large HDL-C showed a positive linear relationship with all-cause mortality and cardiovascular disease mortality [31]. More and more evidence has suggested that the biological function of lipoprotein particles, particularly HDL particles, could differ by size [32]. Total HDL-C has been reported to have a U-shaped (or J-shaped) dose-response relationship with all-cause and cause-specific mortality in the general population and individuals with coronary artery disease [33–35]. The above findings suggest that total HDL-C does not independently reflect the conformation and function of high-density HDL. So, the size of the lipoprotein particles may need to be considered when analysing and interpreting the relationship between lipid composition and renal function.

To our current understanding, this is the initial instance that MR analysis has been employed to explore the intricate connections between SGLT2 inhibition, HDL, and renal function in the general population. Furthermore, we present genetic evidence that suggests a potential mechanism of action for SGLT2 inhibition. This mechanism appears to enhance renal function by modifying HDL concentrations. In order to obtain robust results, we selected three renal function-related indicators (eGFR, UACR, and albuminuria) as outcome variables, In this MR study on renal function outcomes, the analysis of SGLT2 inhibitors showed a consistent trend. Hence, this research offers the advantage of more accurately estimating causal relationships while minimising the influence of confounding biases when compared to traditional observational studies.

However, it is essential to acknowledge that the study has some limitations. Initially, the majority of subjects in the GWAS were of European descent. Therefore, the generalizability of our findings to other ethnic groups and regions requires further validation. Secondly, SGLT2 inhibition is primarily targeted at a single therapy involving the SLC5A2 gene. The lack of a sufficient number of genetic variations linked to the expression levels of SLC5A2 poses a challenge to attaining a desirable degree of accuracy for the MR study [36]. Consequently, we selected six genetic variants that were strongly linked to SLC5A2 and HbA1c expression levels. Additionally, it is important to consider that the drug formulation, dosage, and mode of administration of SGLT2 inhibition may impact their effects on renal function. Therefore, further studies are required to explore this aspect in more detail in the future.

## Conclusion

In conclusion, detailed tests and analysis have shown that genetic predictors of SGLT2 inhibition are linked to HDL and renal function. It also showed that HDL particle concentration is crucial to SGLT2 inhibition’s renal function protection. These findings offer fresh insights into how SGLT2 inhibition reduces renal disease risk and offer clinical practice recommendations for its better use.

### Electronic supplementary material

Below is the link to the electronic supplementary material.

Supplementary Material 1

Supplementary Material 2

Supplementary Material 3

Supplementary Material 4

Supplementary Material 5

## Data Availability

This study uses publicly available CKDGen GWAS Summary statistics (https://ckdgen.imbi.uni-freiburg.de/), IEU OpenGWAS project (https://gwas.mrcieu.ac.uk/), the GTEx Portal (https://www.gtexportal.org/), the eQTLGen Consortium (https://eqtlgen.org/) and the DIAGRAM consortium (https://diagram-consortium.org/). The details are listed in the Supplementary file.
